# Dynamics of Drone Blades Based on Polymer Nanocomposites Incorporating Graphene, Carbon Nanotube, and Fullerene

**DOI:** 10.3390/polym18060778

**Published:** 2026-03-23

**Authors:** Workineh G. Gomera, Tomasz Tański, Jung Yong Kim

**Affiliations:** 1 Department of Materials Science and Engineering, Adama Science and Technology University, Adama 01888, Ethiopia; workineh.gebeyehu@astu.edu.et; 2 Institute of Engineering Materials and Biomaterials, Faculty of Mechanical Engineering, Silesian University of Technology, 44-100 Gliwice, Poland; 3 Department of Renewable Energy, Korean Institute of Technology and Culture, Samarkand International University of Technology, Samarkand 140100, Uzbekistan

**Keywords:** dynamics, nanocomposite, drone blade, graphene, carbon nanotube, fullerene, nanocarbons, finite element method, classical laminate plate theory

## Abstract

Polymer nanocomposites offer significant potential for improving the strength-to-weight ratio and dynamic behavior of drone blades. This study examines the vibration characteristics of tapered aramid (Kevlar)/epoxy composite blades reinforced with nanocarbon fillers—graphene (2D), multi-walled carbon nanotubes (MWCNTs, 1D), and fullerene (0D)—to determine the most effective filler for enhancing stiffness and operational stability. The laminated blades (300 mm length, 200 mm width, root thickness 13 mm, tip thickness 8 mm) incorporate ply drop-offs and a central honeycomb core. Modeling was performed using classical laminate plate theory integrated with the finite element method (FEM) in MATLAB (R2016a). Under clamped–free–free–free boundary conditions, the study considered rotational speeds of 750–2250 rpm, setting angles of 30–60°, various fiber orientations, and nanofiller contents of 0–10 wt.%. The results indicate that while the setting angle minimally affects natural frequency, it significantly influences damping in modes (1,2) and (2,1). Increasing nanofiller content improves stiffness, with optimal performance observed near 5 wt.%. At 1500 rpm in mode (1,1), MWCNTs provided the greatest enhancement. Overall, MWCNTs exhibited superior stiffness improvement and rotational stability compared to other fillers.

## 1. Introduction

Significant research has been conducted to optimize the structural components of unmanned aerial vehicles (UAVs), also referred to as drones, for improved performance, durability, and efficiency due to the rapid evolution of these vehicles [1,2,3,4,5,6,7,8]. Drone blades, also known as propellers, are essential for thrust production (mechanical force), aerodynamic performance, and overall flight stability [3,4,5,6,7,8]. A possible approach to enhancing the mechanical characteristics, weight efficiency, and resilience of drone blades is the development of nanocomposite materials such as carbon fiber-reinforced polymer (CFRP), glass fiber-reinforced polymer (GFRP), Kevlar (aramid), and carbon-reinforced nylon [9,10,11]. Furthermore, laminar composites such as balsawood, fiberglass, polyester resin, CFRP reinforced with continuing fibers, E-glass/epoxy, Kevlar/epoxy, and carbon/epoxy can be used as UAV wing structure materials [12]. Specifically, the high-strength aramid fiber is well known for its durability, impact resistance, and lightweight, all of which enhance graphene’s qualities in hybrid composites [13]. It is possible to obtain a superior strength-to-weight ratio, increased fatigue resistance, and improved durability under dynamic loading conditions when one uses a composite material for drone blades [14,15].

The ability of composite materials to sustain quasi-static and dynamic stresses while preserving structural integrity has been investigated for their application in UAVs [16,17,18]. Drone blades operating in tough conditions require exceptional energy absorption and impact damage resistance, which Kevlar-reinforced composites have proven to offer [19]. For example, in the case of Kevlar/nanocarbon (graphene) composite, Kevlar contributes to enhanced toughness and flexibility, whereas graphene increases stiffness and thermal stability [20]. Additionally, 3D printing and other additive manufacturing techniques have made it easier to precisely fabricate intricate composite structures, allowing for customized drone blade designs with the best possible mechanical and aerodynamic performance [21]. Research on improved composites for aerospace applications has examined how composite drone blades behave structurally under different stress scenarios, emphasizing the significance of production procedures and material selection [8]. As an illustration, when Kevlar was mixed with carbon fiber and glass fiber, the composite could show high strength and lightweight characteristics [22]. This is because Kevlar fiber has excellent mechanical properties, e.g., tensile strength ~3.6 GPa and modulus ~120 GPa. Thus, it has been widely used in the aircraft industry, military gear, the automotive industry, and sporting equipment [23,24,25,26].

Graphite has a stacked planar sp_2_-hybridized C_6_ ring hexagonal structure [27]. Herein, if each layer is separated, a single planar sheet could be called graphene with one atom thickness in a 2D-layered hexagonal lattice [28]. Interestingly, if it is rolled up, it transforms into a carbon nanotube (CNT) with a 1D cylindrical structure, whereas if it rolls into a ball, it is called fullerene with a 0D spherical structure (hollow cage), like C_60_ or C_70_ [29]. To date, graphene and CNT have been frequently incorporated into Kevlar–fiber-based composites [30,31,32,33,34,35,36,37,38], whereas fullerene has not been tested for Kevlar composites, although all of them are carbon allotropes. At this moment, it should be pointed out that when a nanofiller (equivalent to oligomer or polymer) is incorporated into a polymer matrix, there is a dispersion (or miscibility) issue depending on the intermolecular interaction and entropy [39,40].

In this study, we investigate theoretically the dynamics of nanocomposite drone blades prepared from Kevlar–epoxy/graphene, Kevlar–epoxy/multi-walled carbon nanotube (MWCNT), and Kevlar–epoxy/fullerene. Herein, we assume that each nanofiller is well dispersed (i.e., without aggregation) in a Kevlar–epoxy matrix. Then, we focus on the mechanical properties, aerodynamic efficacy, and endurance of nanocomposites under operational conditions. Importantly, considering that there is no report regarding Kevlar/fullerene composite as far as the author’s knowledge, our report should be important in the field of Kevlar-based nanocomposites. Furthermore, considering the versatile dimension of nanocarbon (2D graphene, 1D MWCNT, and 0D fullerene), this study might provide some insight regarding the shape of the nanofiller in a polymer matrix. Herein, based on classical laminated plate theory (CLPT) [41], the governing differential equations of motion of the individual plates of a rotating thickness tapered laminated composite plate are obtained and presented in a finite element formulation [42] that takes into account the various rotational effects. Specifically, we describe the efficacy of the created finite element formulation by comparing the natural frequencies and damping ratios.

## 2. Theoretical Methods

**Figure 1:**
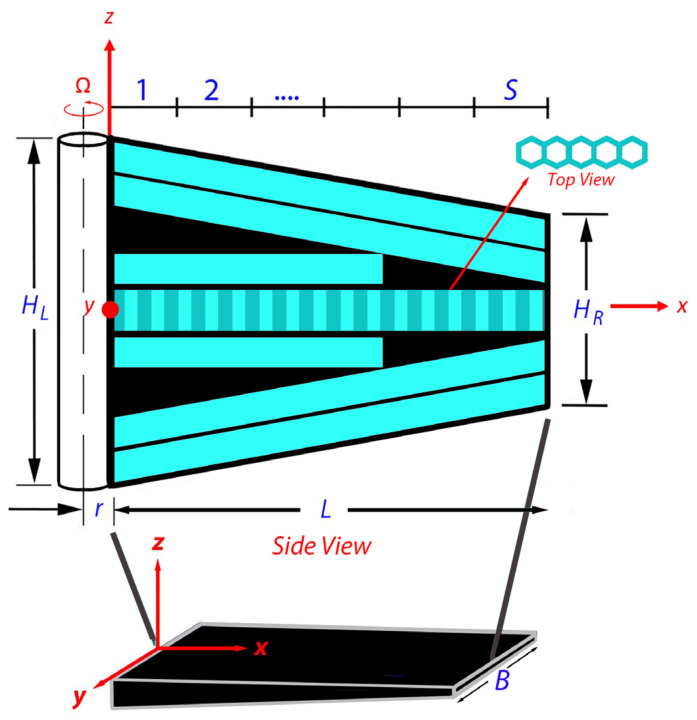
Tapered plate representation of rotating composite blades for drones. *H*_L_ = 13 mm, *H*_R_ = 8 mm, *r* = 2 mm, *L* = 300 mm, and *B* = 200 mm. The top (or bottom) layer is composed of three laminates with a thickness of 1.25 mm, whereas the honeycomb core layer is 3 mm thick.

Figure 1 shows a tapered laminated composite plate, formed by internally dropping off and altering the plies, which is considered to formulate the numerical model. The thickness *H*_L_ = 13 mm and *H*_R_ = 8 mm are the heights at the left and right ends of the plate. Here, it is notable that these height thicknesses are relatively small compared to the length *L* = 300 m and width = 200 mm of the composite plate. In order to formulate a linearly varying taper section along the longitudinal direction *x*, the taper plates are divided into *S* number of domains along the longitudinal direction. Furthermore, the top and bottom layers are composed of three plies (each ply is 1.25 mm thick), respectively, whereas the center layer is one single honeycomb layer, an array of hexagonal pillars.

**Figure 2:**
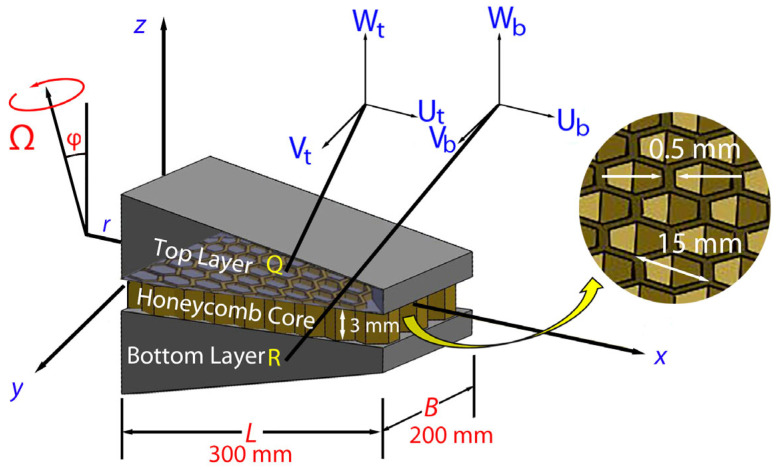
Schematics of a tapered plate composed of a top layer, honeycomb core, and bottom layer. Here, *L* and *B* denote the length and width of the tapered plate, respectively. Ω and *φ* are the angular velocity and rotation angle, respectively. Honeycomb dimension: cell size = 15 mm, cell wall thickness = 0.5 mm, and core height = 3 mm.

The tapered laminated composite plate is assumed to rotate with a constant angular velocity $\vec{\Omega }=\Omega_{x}\vec{i}+\Omega_{y}\vec{j}+\Omega_{z}\vec{k}$ about an axis that lies along the *y–z* plane, as shown in Figure 2. Here, $\Omega_{x}$, $\Omega_{y}$, and $\Omega_{z}$ denote the angular velocity components about the *x*-, *y*-, and *z*-axis, respectively. Since the orientation of the plate is confined to the *y–z* plane, only the angular velocity components $\Omega_{y}=\Omega \sin\varphi$ and $\Omega_{z}=\Omega \cos\varphi$ are considered because $\Omega_{x}=0$. Furthermore, the φ value is the inclination angle between the angular velocity vector and the reference axis of the plate.

Strain energy represents the elastic potential energy stored in a deformed material (change in shape or size), recoverable upon unloading, often calculated via stiffness matrices in structures. It is composed of linear and rotational strain energy. The former results from axial (tension/compression) or bending forces, whereas the latter is from twisting moments. On the other hand, kinetic energy encompasses linear motion and rotation, combining in rigid body dynamics for total mechanical energy conservation. First, based on CLPT [41], the linear strain energy ($U_{m,b}$) associated with membrane and bending deformations can be expressed as follows:

(1) $$U_{m,b}=\frac{1}{2}\int_{0}^{L}\int_{-\frac{B}{2}}^{\frac{B}{2}}{\left\{\begin{array}{c} \frac{\partial u^{0}}{\partial x}\\ \frac{\partial v^{0}}{\partial y}\\ \frac{\partial u^{0}}{\partial y}+\frac{\partial v^{0}}{\partial x}\\ -\frac{\partial^{2}w^{0}}{\partial x^{2}}\\ -\frac{\partial^{2}w^{0}}{\partial y^{2}}\\ -2\frac{\partial^{2}w^{0}}{\partial x\partial y} \end{array}\right\}}^{T}\left[\begin{array}{cc} \begin{array}{ccc} A_{11}(x) & A_{12}(x) & A_{16}(x)\\ A_{12}(x) & A_{22}(x) & A_{26}(x)\\ A_{16}(x) & A_{26}(x) & A_{66}(x) \end{array} & \begin{array}{ccc} B_{11}(x) & B_{12}(x) & B_{16}(x)\\ B_{12}(x) & B_{22}(x) & B_{26}(x)\\ B_{16}(x) & B_{26}(x) & B_{66}(x) \end{array}\\ \begin{array}{ccc} B_{11}(x) & B_{12}(x) & B_{16}(x)\\ B_{12}(x) & B_{22}(x) & B_{26}(x)\\ B_{16}(x) & B_{26}(x) & B_{66}(x) \end{array} & \begin{array}{ccc} D_{11}(x) & D_{12}(x) & D_{16}(x)\\ D_{12}(x) & D_{22}(x) & D_{26}(x)\\ D_{16}(x) & D_{26}(x) & D_{66}(x) \end{array} \end{array}\right]\left\{\begin{array}{c} \frac{\partial u^{0}}{\partial x}\\ \frac{\partial v^{0}}{\partial y}\\ \frac{\partial u^{0}}{\partial y}+\frac{\partial v^{0}}{\partial x}\\ -\frac{\partial^{2}w^{0}}{\partial x^{2}}\\ -\frac{\partial^{2}w^{0}}{\partial y^{2}}\\ -2\frac{\partial^{2}w^{0}}{\partial x\partial y} \end{array}\right\}\;dxdy\;$$

where the two subscripts in the left-hand side indicate membrane (*m*) and bending (*b*), respectively. $u^{o}$ and $v^{o}$ are the mid-plane displacement in *x* and *y* out-of-plane displacement, respectively, whereas $w^{0}$ is that in the z-direction. The elements $A_{ij}(x)$, $B_{ij}(x)$, and $D_{ij}(x)$ are the components of the extensional stiffness matrix, coupling matrix, and bending stiffness matrix, respectively. Derivation of each element of the matrix can be found in Appendix A (see Equations (A1)–(A3)). Second, the rotational strain energy ($U_{r}$) can be defined as follows:

(2) $$U_{r}=\frac{1}{2}\int_{0}^{L}\int_{-\frac{B}{2}}^{\frac{B}{2}}{\left\{\begin{array}{c} \frac{\partial w^{0}}{\partial x}\\ \frac{\partial w^{0}}{\partial y} \end{array}\right\}}^{T}\left[\begin{array}{cc} N_{x}^{r}(x) & 0\\ 0 & N_{y}^{r}(x,y) \end{array}\right]\left\{\begin{array}{c} \frac{\partial w^{0}}{\partial x}\\ \frac{\partial w^{0}}{\partial y} \end{array}\right\}dxdy$$

where $N_{x}^{r}(x)$ and $N_{y}^{r}(x,y)$ are the normal force per unit width in the *x-* and *y*-directions, respectively. Third, the kinetic energy contributions arising from inertial effects $T_{i}$, Coriolis forces $T_{c}$, and displacement-dependent centrifugal forces $T_{a}$, associated with the axial deformations and transverse deflection of a rotating tapered laminated composite plate, can be expressed as follows:

(3) $$T_{i}=\frac{1}{2}\int_{0}^{L}\int_{-\frac{B}{2}}^{\frac{B}{2}}\int_{-\frac{H}{2}}^{\frac{H}{2}}{\left\{\begin{array}{c} {\dot{u}}^{0}\\ {\dot{v}}^{0}\\ {\dot{w}}^{0}\\ \frac{\partial {\dot{w}}^{0}}{\partial x}\\ \frac{\partial {\dot{w}}^{0}}{\partial y} \end{array}\right\}}^{T}{\left[\begin{array}{ccccc} \rho & 0 & 0 & -\rho \;z(x) & 0\\ 0 & \rho & 0 & 0 & -\rho \;z(x)\\ 0 & 0 & \rho & 0 & 0\\ -\rho \;z(x) & 0 & 0 & \rho \;z^{2}(x) & 0\\ 0 & -\rho \;z(x) & 0 & 0 & \rho \;z^{2}(x) \end{array}\right]}^{T}\;\left\{\begin{array}{c} {\dot{u}}^{0}\\ {\dot{v}}^{0}\\ {\dot{w}}^{0}\\ \frac{\partial {\dot{w}}^{0}}{\partial x}\\ \frac{\partial {\dot{w}}^{0}}{\partial y} \end{array}\right\}\;dxdydz$$

(4) $$T_{c}=\frac{1}{2}\int_{0}^{L}\int_{-\frac{B}{2}}^{\frac{B}{2}}\int_{-\frac{H}{2}}^{\frac{H}{2}}{\left\{\begin{array}{c} {\dot{u}}^{0}\\ {\dot{v}}^{0}\\ {\dot{w}}^{0} \end{array}\right\}}^{T}\left[\begin{array}{ccc} 0 & -2\rho \;\Omega_{z} & 2\rho \;\Omega_{y}\\ 2\rho \;\Omega_{z} & 0 & -2\rho \;\Omega_{x}\\ -2\rho \;\Omega_{y} & 2\rho \;\Omega_{x} & 0 \end{array}\right]\left\{\begin{array}{c} u^{0}\\ v^{0}\\ w^{0} \end{array}\right\}\;dxdydz\;$$

(5) $$\;T_{a}=\frac{1}{2}\int_{0}^{L}\int_{-\frac{B}{2}}^{\frac{B}{2}}\int_{-\frac{H}{2}}^{\frac{H}{2}}{\left\{\begin{array}{c} u^{0}\\ v^{0}\\ w^{0} \end{array}\right\}}^{T}\left[\begin{array}{ccc} \rho \;(\Omega_{y}^{2}+\Omega_{z}^{2}) & -\rho \;\Omega_{x}\Omega_{y} & -\rho \;\Omega_{x}\Omega_{z}\\ -\rho \;\Omega_{x}\Omega_{y} & \rho \;(\Omega_{x}^{2}+\Omega_{z}^{2}) & -\rho \;\Omega_{z}\Omega_{y}\\ -\rho \;\Omega_{x}\Omega_{z} & -\rho \;\Omega_{z}\Omega_{y} & \rho \;(\Omega_{x}^{2}+\Omega_{y}^{2}) \end{array}\right]\left\{\begin{array}{c} u^{0}\\ v^{0}\\ w^{0} \end{array}\right\}\;dxdydz$$

where Ω and ρ are the angular speed about the local axis and the density of material, repetitively.

The finite element method (FEM) [42] is essential for solving problems in tapered plate composite materials research. This is because these composite structures feature variable thickness via ply drop-offs, anisotropic material properties from layered fiber orientations, geometric nonlinearity, and complex boundary/loading conditions. It should be pointed out that the analytical solutions are rarely feasible due to the intricate coupling of bending, shear, torsion, and inter-laminar stresses, along with discontinuities at taper locations, causing stress concentrations and potential delamination. The FEM approach discretizes the domain into elements, accurately capturing these heterogeneities, variable stiffness, mode shapes, natural frequencies, and nonlinear dynamic behaviors, while it enables parametric studies on taper ratios, fiber angles, nanofiller reinforcements, and rotational effects. A 4-noded quadrilateral isoparametric plate element is used. This is standard for CLPT-based models of laminated plates, particularly tapered designs with ply drop-offs. While the literature [43] frequently uses these elements formulated with first-order shear deformation theory, this model specifically accounts for the thickness relative to in-plane dimensions [300 × 200 mm^2^] to accurately capture bending, shear, and inter-laminar effects at the drop-offs.

Thus, the FEM process provides reliable predictions of stress distributions, failure modes, and performance optimization. The reader can find the FEM formulation for stiffness, Coriolis, and mass matrices in Appendix A (see Equations (A8)–(A14)). Furthermore, it is important to note that the physical parameters for each material used in this study can be found in Table 1. Note that more information can be found in the Supplementary Materials.

**Custom Implementation in MATLAB**:

The element is user-coded based on classical laminate theory extensions for variable thickness. The stiffness matrix, mass matrix, and Coriolis matrix are assembled per Equations (A8)–(A14) in Appendix A, following the standard isoparametric formulation with bilinear shape functions.

Dimensions (nodal degrees of freedom per element):

- Four nodes per element (quadrilateral);
- Five or six degrees of freedom per node (typical for plate elements in such analyses).

## 3. Results and Discussion

**Figure 3:**
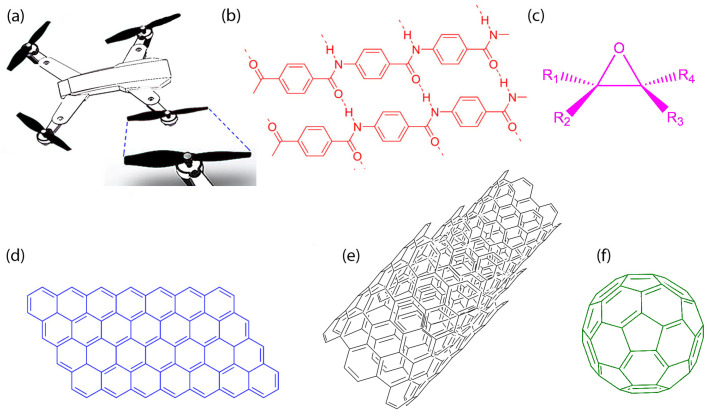
(**a**) Schematic drawing of a drone and its blade. Chemical structures of (**b**) aramid, (**c**) epoxy, (**d**) graphene, (**e**) multi-walled carbon nanotube, and (**f**) fullerene.

Figure 3a shows a schematic drawing of a drone and its blade. In this study, the composite for drone blades was made by an aramid/epoxy composite incorporating nanocarbons. Figure 3b–f exhibit the chemical structures of (b) aramid, (c) epoxy, (d) graphene, (e) multi-wall carbon nanotube, and (f) fullerene. Interestingly, aramid is a fully aromatic polymer with the commercial name Kevlar, with superior mechanical properties [19]. Hence, Kevlar could be an excellent candidate as a polymer matrix for composite blades. On the other hand, epoxy has unique advantages in adhesion, mechanical properties, chemical resistance, and others [51]. Hence, in this study, we use Kevlar–epoxy as a composite polymer matrix, i.e., continuous binder phase for accommodating nanofillers.

Herein, the composite polymer matrix without nanofiller is composed of a Kevlar:epoxy = 60:40 weight ratio. In this matrix, when we add a 10% nanofiller, the composite will have the composition of Kevlar:epoxy:nanocarbon = 55:35:10 (via deducting equally 5% from both Kevlar and epoxy). In this process, the rule of mixtures can be elucidated by Equations (6)–(11) [52,53]. The longitudinal modulus ($E_{1}$) in fiber direction is defined as below.

(6) $$E_{1}=V_{f}E_{f}+V_{m}E_{m}$$

where $V_{f}$ and $V_{m}$ are the volumetric ratios of fiber and matrix, whereas $E_{f}$ and $E_{m}$ are the elastic modulus of the fiber and matrix, respectively. On the other hand, the transverse modulus ($E_{2}$) perpendicular to fiber direction is defined as below.

(7) $$E_{2}=\frac{E_{f}E_{m}}{V_{f}E_{f}+V_{m}E_{m}}$$

The elastic modulus (η) of a nanocomposite material is defined as follows:

(8) $$\eta =\frac{\left(\frac{E_{n}}{E_{m}}\right)-1}{\left(\frac{E_{n}}{E_{m}}\right)+\zeta }$$

where ζ is a shape function and $E_{n}$ is the modulus of the nanofiller. The effective modulus ($E_{m,eff}$) of the nanofiller-reinforced composite matrix is

(9) $$E_{m,eff}=E_{m}\frac{1+\zeta \eta V_{n}}{1-\eta V_{n}}$$

where $V_{n}$ is the volumetric ratio of the nanofiller. The shear modulus (G) of the fiber–matrix ratio is

(10) $$G=\frac{G_{f}G_{m}}{V_{f}G_{m}+V_{m}G_{f}}$$

where $G_{m}$ and $G_{f}$ are the shear modulus of the matrix and fiber, respectively. The density of the composite ($\rho_{c}$) is expressed as follows:

(11) $$\rho_{c}=V_{f}\rho_{f}+V_{m}\rho_{m}+V_{n}\rho_{n}$$

where $\rho_{f}$, $\rho_{m}$, and $\rho_{n}$ are the densities of the fiber, matrix, and nanofiller, respectively.

In this study, using MATLAB software (version 2016a), we analyzed the dynamic properties of Kevlar–epoxy/nanocarbon composites. Herein, the natural frequency (also called inherent or intrinsic frequency) refers to the rate at which a system oscillates after an initial disturbance when it is not subjected to a continuous external force. It depends on the stiffness, mass distribution, boundary conditions, geometry, and material properties. On the other hand, the damping ratio is a dimensionless parameter that quantifies how quickly vibration amplitude (oscillation) decays after being disturbed. It represents the energy dissipation capability of the composite plate due to mechanisms such as matrix viscoelasticity, interfacial friction between the nanofiller and matrix, microcracking, and internal friction. The study of natural frequency and damping ratio is crucial for understanding, predicting, and controlling the dynamic behaviour of composite plates. This is because they play a critical role in safe design, performance optimization, vibration mitigation, and long-term durability of composite structures under dynamic loading. Specifically, we used clamped–free–free–free (CFFF) as a boundary condition in the dynamic analysis of mechanics when nanofillers are varied from 0 to 10 weight %.

**Figure 4:**
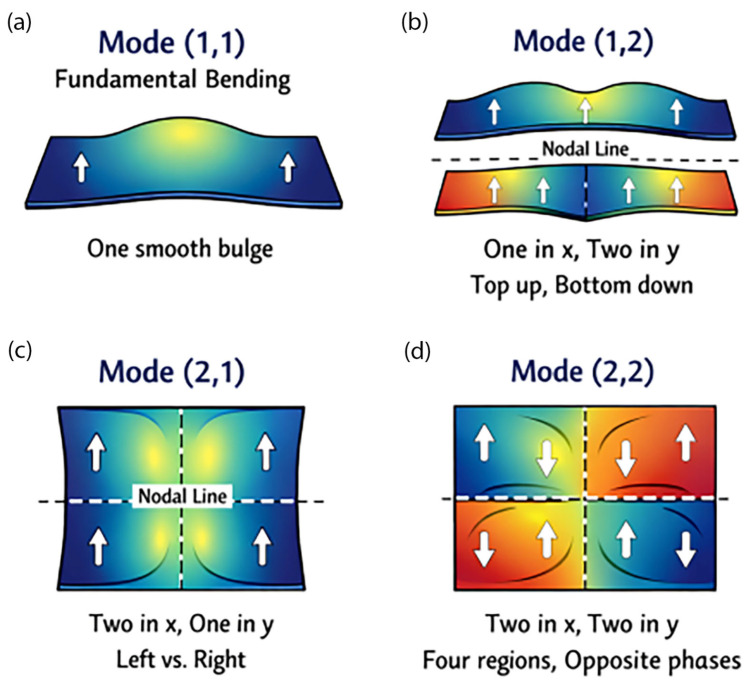
Mode shape: (**a**) Mode (1,1); (**b**) Mode (1,2); (**c**) Mode (2,1); and (**d**) Mode (2,2).

Furthermore, a mode shape (Figure 4) shows us how the plate vibrates at a specific natural frequency. Each mode–number pair explains how many half-waves appear along the *x*- and *y*-directions. Mode (1,1) is a fundamental bending mode where the entire plate goes up and down in one smooth bulge, with no internal nodal lines. Mode (1,2) is one wave in the *x*-direction and two waves in the *y*-direction, where the plate bends like two long strips stacked vertically. There is one nodal line across the middle, and the top half moves up while the bottom half moves down (opposite directions). Mode (2,1) is two waves in the *x*-direction and one wave in the *y*-direction, where the latter bends like two side-by-side strips with one vertical nodal line down the center. Herein, the left and right sides vibrate opposite each other. Mode (2,2) is two waves in the *x*-direction and two waves in the *y-*direction, where the plate has four vibrating regions. In this mode, adjacent quadrants vibrate in opposite directions, and there is a higher frequency because the shape is more complex. In this study, the effect of nanocarbon in the composite on the variation of the transverse vibration mode shape is investigated in detail as follows.

**Figure 5:**
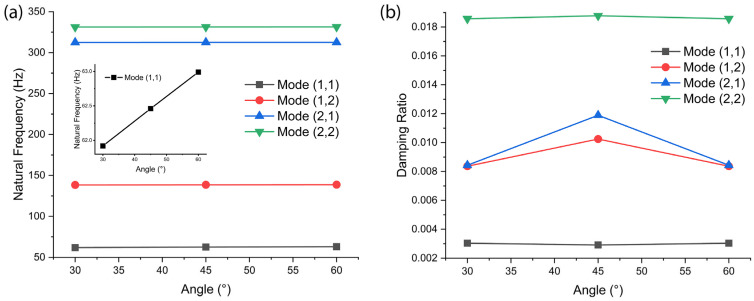
(**a**) Natural frequency and (**b**) damping ratio of graphene (5 wt.%)-reinforced Kevlar–epoxy composite as a function of setting angle (*φ* = 30°, 45°, and 60°) at fiber orientation-1 (FO-1) [90°/0°/90°] and rotating speed (=2250 rpm). Inset of (**a**) magnification of Mode (1,1) regarding natural frequency vs. setting angle.

As a first step, before comparing three nanocarbons, we studied the effect of vibration mode type and fiber orientation on the natural frequency of graphene-reinforced Kevlar–epoxy composite as a model system. Figure 5 shows (a) the natural frequency and (b) the damping ratio of graphene (5 wt.%)-reinforced Kevlar–epoxy composite as a function of setting angle (*φ* = 30°, 45°, and 60°). Herein, the natural frequencies were obtained using the energy method based on the Rayleigh–Ritz formulation [54]. The linear strain energy, rotational strain energy, and kinetic energy expressions are presented in Section 2. Theoretical methods detailed in Appendix A were substituted into the governing eigenvalue equation derived from Hamilton’s principle (Equations (1) and (A16)) [55]. Applying the variational procedure yields the matrix eigenvalue problem, and the resulting characteristic equation was solved to determine the fundamental and higher-mode natural frequencies. Additionally, we have clarified that the damping ratio was determined using the equivalent viscous damping model, incorporating material damping contributions from the polymer matrix, fiber reinforcement, and nanofiller phase through the complex modulus approach (Equations (7), (8), and (A1)–(A16)) [54,55].

As shown in Figure 5a, the natural frequency is almost constant. However, as magnified in the Figure 5a inset, it increases linearly with increasing setting angle. On the other hand, the damping ratio (Figure 5b) shows a different behavior by showing the maximum value at the setting angle of 45° for Mode (1,2) and Mode (2,1). In the case of Mode (1,1) and Mode (2,2), the minimum and maximum were observed at 45°, respectively, although the change in the damping ratio is negligibly small. Hence, based on Figure 5, we conclude that the setting angle affects the natural frequency very slightly, whereas it affects the damping ratio significantly for Modes (1,2) and (2,1). Note that for this calculation, we used Equation (A7) in Appendix A.

**Figure 6:**
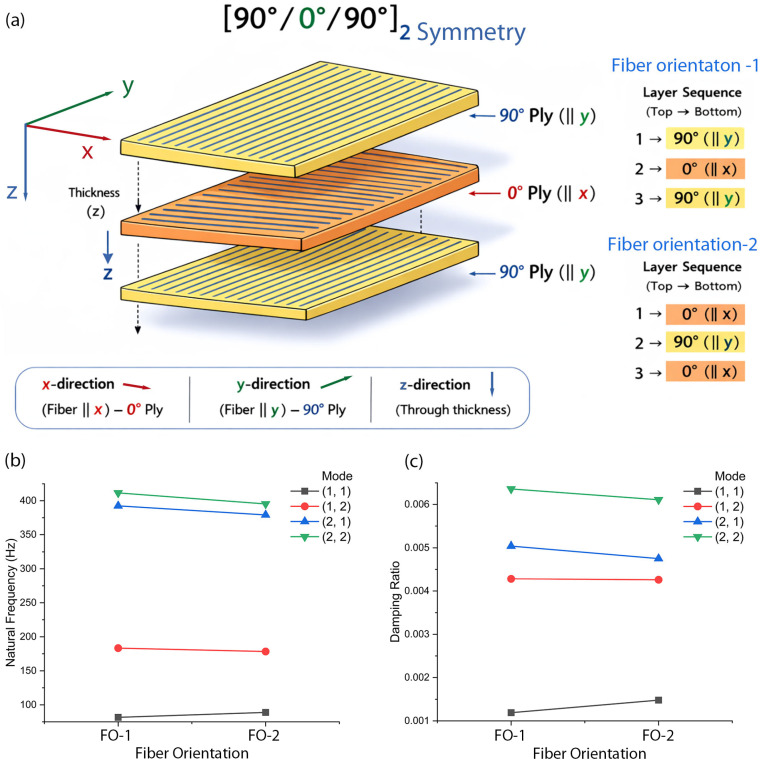
(**a**) Fiber orientation-1 [90°/0°/90°] and fiber orientation 2 [0°/90°/0°]. (**b**) Natural frequency of graphene (5 wt.%)-reinforced Kevlar–epoxy composite as a function of fiber orientation for each mode at setting angle (*φ* = 45°) and rotating speed (=2250 rpm). (**c**) Damping ratio as a function of fiber orientation for each mode at setting angle (*φ* = 45°) and rotating speed (=2250 rpm).

Figure 6a shows two types of fiber orientation (FO). When the top three laminate layers are composed of the fiber orientation [90°/0°/90°], we call it FO-1. Here, 90° and 0° indicate that the main chain of aramid is roughly oriented to the direction of the *y*-axis and the *x*-axis, respectively, as shown in Figure 6. Recall that the top and bottom layers are symmetric, indicating the bottom layer has also [90°/0°/90°] orientation if the top layer has FO-1. Similarly, in the case of FO-2, the fiber orientation is FO-1 [0°/90°/0°]. Figure 6b shows the natural frequency as a function of FO. In Mode (1,1), the natural frequency is slightly higher in FO-2 compared to FO-1, whereas in the other three modes, it is slightly higher in FO-1. Interestingly, the damping ratio graph in Figure 6c shows a similar trend to that observed in Figure 6b. Note that the Equations (A3)–(A7) in Appendix A were used for this calculation.

**Figure 7:**
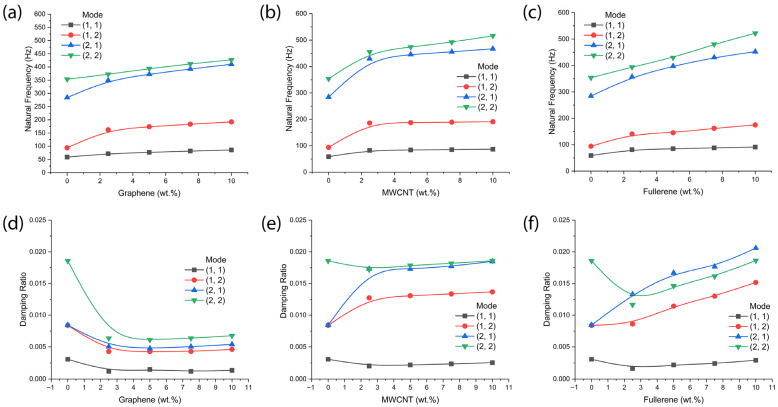
Natural frequency as a function of nanocarbon weight percent at fiber orientation (FO-1), rotating speed (=2250 rpm), and setting angle (*φ* = 45°): (**a**) graphene, (**b**) MWCNT, and (**c**) fullerene- reinforced Kevlar–epoxy composite. Damping ratio as a function of nanocarbon weight percent: (**d**) graphene, (**e**) MWCNT, and (**f**) fullerene-reinforced Kevlar–epoxy composite.

Through Figure 5 and Figure 6, we first demonstrated that both the setting angle and the aramid fiber orientation in the ply affected the natural frequency and damping ratio. Next, we investigated the effect of nanocarbon species on the dynamic properties of the composite at the fixed conditions of FO-1, *φ* = 45°, and rotating speed of 2250 rpm. Figure 7a–c show the natural frequency as a function of the weight percent of each nanocarbon. As shown in Figure 7a–c, the overall trend (i.e., the enhanced natural frequency with increasing nanocarbon amounts compared to pure aramid–epoxy composites without nanocarbon) was similarly observed, although the degree of enhancement was different. However, at 2250 rpm, MWCNT (Figure 7b) looks best among the three samples.

Several experimental studies on Kevlar/epoxy nanocomposites reinforced with nanocarbon fillers support the stiffness enhancements predicted by the current model. For graphene nanoplatelet additions, dynamic mechanical analysis has shown increased storage moduli and modified viscoelastic behavior, indicating improved interfacial stress transfer [56]. Modal investigations on graphene/epoxy composites further revealed significant increases in damping ratios—up to ~50% at moderate loading—though excessive filler content may reduce natural frequencies due to added mass effects [57]. Similarly, MWCNT-reinforced epoxy composites demonstrated a 5–10% increase in fundamental natural frequency, confirming the stiffness-driven enhancements predicted by effective modulus models [58]. However, damping behavior remains highly dependent on dispersion quality and interfacial bonding; some studies even report reduced damping due to constrained micro-slippage [58]. In contrast, fullerene (C60) modifications yielded only modest improvements in static mechanical properties with limited vibration data. This is consistent with theoretical expectations that spherical nanofillers provide lower stiffness gains than high-aspect-ratio graphene or nanotubes [59].

Figure 7d–f show the damping ratio as a function of the weight percent of each nanocarbon. As shown in Figure 7d, the graphene-reinforced composite material shows a depression of the damping ratio with increasing graphene amounts at all the modes from (1,1) to (2,2). However, in the case of MWCNT and fullerene, they show the enhanced damping ratio when the mode is (1,2) or (2,1), indicating the complexity of the characteristics of composite materials. Hence, we studied the same composite system at different rotating speeds, such as 750 rpm and 1500 rpm. The results were displayed in Table A1, Table A2, Table A3, Table A4, Table A5, Table A6, Table A7, Table A8, Table A9, Table A10, Table A11 and Table A12 in Appendix A. For example, at 5 wt.% of nanocarbon in composite blades, the natural frequency was 74.4 Hz for graphene, 76.5 Hz for MWCNT, and 68.2 Hz for fullerene, respectively, at the medium rotating speed of 1500 rpm under Mode (1,1). This result indicates that the natural frequency was enhanced by 21.0%, 24.4%, and 10.9%, respectively, based on the aramid–epoxy’s inherent frequency of 61.5 Hz. However, as shown in Figure 7a–c, at 5 wt.% of nanocarbon, the natural frequency was 76.9 Hz for graphene, 84.1 Hz for MWCNT, and 84.5 Hz for fullerene at the high rotating speed of 2250 rpm under Mode (1,1). This result suggests that the natural frequency was enhanced by 30.3%, 42.5%, and 43.2%, respectively, based on the polymer composite’s inherent frequency of 59.0 Hz. Hence, the stiffness behavior is dependent on the input parameter condition, e.g., the rotating speed of the composite plate. Furthermore, these findings provide insights and encourage further study on the material properties as a function of rotating speed.

**Figure 8:**
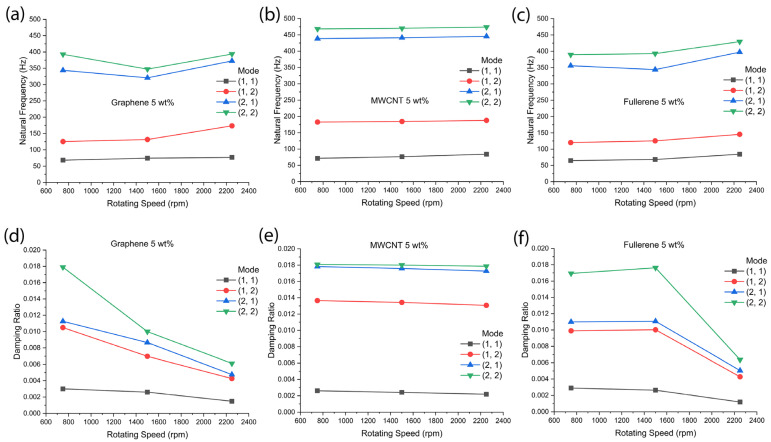
Natural frequency as a function of rotating speed at fiber orientation (FO-1) and setting angle (*φ* = 45°): (**a**) graphene, (**b**) MWCNT, and (**c**) fullerene-reinforced Kevlar–epoxy composite. Damping ratio as a function of rotating speed: (**d**) graphene, (**e**) MWCNT, and (**f**) fullerene-reinforced Kevlar–epoxy composite.

We studied the effect of rotating speed on mechanical properties. Figure 8 shows the natural frequency and damping ratio as a function of rotating speed for the nanocarbon-reinforced aramid–epoxy composite materials. First, in Figure 8a–c, the most striking observation is the behavior of the MWCNT-based nanocomposite. As shown in Figure 8b, the natural frequency of the MWCNT-based system is very stable and high compared with the other data of graphene- or fullerene-based nanocomposites. Second, when we see the blue-filled triangle data at Mode (2,1) in Figure 8a,c, the natural frequency could partially decrease with increasing rotating speed, e.g., from 750 rpm to 1500 rpm. On the other hand, Figure 8d–f show the damping ratio as a function of rotating speed. First, the most striking observation is that the damping ratio is almost stable in the MWCNT-reinforced composite system. Second, with increasing rotating speed, the graphene-reinforced composite shows an almost monotonous decrease. Third, in the case of fullerene-reinforced composite, the damping ratio is stable from 750 rpm to 1500 rpm, but it decreased when the rotating speed was increased up to 2250 rpm. Hence, based on Figure 8, the MWCNT stands out as a nanofiller among the tested nanocarbons, consistent with previous reports [60,61].

## 4. Conclusions

We investigated the dynamic characteristics of nanocarbon-reinforced aramid–epoxy composites for drone blade applications. For the first time, three carbon allotropes—2D graphene, 1D multi-walled carbon nanotubes (MWCNTs), and 0D fullerene—were compared to pure aramid–epoxy composites without nanocarbon. As a first step, we examined the effects of setting angle on the natural frequency and damping ratio for the four different modes. Then, we found that the setting angle affects the natural frequency very slightly, whereas it affects the damping ratio significantly for Modes (1,2) and (2,1). Second, we investigated the effect of aramid fiber orientation in a ply on the natural frequency and damping ratio. Then, we realized that depending on the mode condition, the natural frequency and damping ratio data trend is different. For example, at Mode (1,1), fiber orientation 2 has a higher value, but for the others, the trend is reversed, although it is a minor difference. Third, at a fixed setting of 45° and a fiber orientation of 1, we compared the performance of graphene, MWCNT, and fullerene as a function of composition. Then, we observed that by increasing the weight fraction of nanocarbon, the natural frequency (stiffness) increased. However, in the case of the damping ratio, the data trend is diverse. For the graphene-reinforced composite, the damping ratio decreases with increasing graphene amount. However, in the case of the MWCNT and fullerene, the damping ratio decreases with increasing nanocarbon in Mode (1,1) and Mode (2,2) but increases in Mode (1,2) and Mode (2,2). Finally, we investigated the effect of rotating speed on the mechanical properties (natural frequency and damping ratio) of the composite. Then, we found that the MWCNT shows a superior stability in both natural frequency and damping ratio, i.e., the data tend is almost level-off independent of the rotating speed. Hence, based on these observations, the MWCNT is the most effective nanofiller for aramid–epoxy composites in lightweight and high-performance drone blade applications. Future work should include complementary experimental studies to account for the actual dispersion behavior of each nanocarbon within the composite matrix, which cannot be fully captured by theoretical models alone.

## Figures and Tables

**Table 1:** Physical parameters of each material in this work.

Materials	Density (g/cm^3^)	Young’s Modulus ^1^ (GPa)	Tensile Strength (GPa)	Ref.
Aramid	1.44	112–124	3.6	[44]
Epoxy	1.15	2.5–3.5	0.05–0.09	[45,46]
Graphene	2.20	1000	130	[47,48]
MWCNT	1.35	270–1200	11–100	[49]
Fullerene	1.65	53–69	-	[50]

^1^ In this study, Young’s moduli used are 83 GPa for aramid, 3.4 GPa for epoxy, 1000 GPa for graphene, 1200 GPa for MWCNT, and 61 GPa for fullerene.

## Data Availability

The original contributions presented in this study are included in the article/Supplementary Materials. Further inquiries can be directed to the corresponding authors.

## Supplementary Materials

The following supporting information can be downloaded at: https://www.mdpi.com/article/10.3390/polym18060778/s1, Geometry and Structural Configuration; Material Properties; Modeling and Numerical Method; Parametric Ranges Studied; Software and Implementation; and The Properties and Dimension of Fibers. Reference [62] is cited in the supplementary materials.

## Abbreviations

The following abbreviations are used in this manuscript:

CFFF	Clamped–free–free–free
CFRP	Carbon fiber-reinforced polymer
CLPT	Classical laminate plate theory
CNT	Carbon nanotube
GFRP	Glass fiber-reinforced polymer
FO	Fiber orientation
MATLAB	Matrix Laboratory
MWCNT	Multi-wall carbon nanotube
ULV	Unmanned aerial vehicle

## Appendix A

To solve the linear strain energy ($U_{m,b}$) associated with membrane and bending deformations, we need to solve the [6 × 6] matrix composed of the elements, $A_{ij}$, $B_{ij}$, and $D_{ij}$ $\left(i,j=1,\;2,\;6\right)$, incorporating the sub-matrix term, [Q], $\left[Q^{\prime \prime }\right]$, $\left[T_{\sigma \varphi }\right]$, and $\left[T_{\sigma \theta }\right]$ [41,63,64,65].

(A1) $$A_{ij}(x)=\int_{-\frac{H}{2}}^{\frac{H}{2}}Q_{ij}\;dz=\sum_{k=1}^{e}\left[{\left(Q_{ij}\right)}_{k}\right]\;((z_{k}+x\tan\theta )-(z_{k-1}+x\tan\theta ))$$

(A2) $$B_{ij}(x)=\int_{-\frac{H}{2}}^{\frac{H}{2}}Q_{ij}\;z\;dz=\frac{1}{2}\sum_{k=1}^{e}\left[{\left(Q_{ij}\right)}_{k}\right]\;({\left(z_{k}+x\tan\theta \right)}^{2}-{\left(z_{k-1}+x\tan\theta \right)}^{2})$$

(A3) $$D_{ij}(x)=\int_{-\frac{H}{2}}^{\frac{H}{2}}Q_{ij}\;z^{2}dz=\frac{1}{3}\sum_{k=1}^{e}\left[{\left(Q_{ij}\right)}_{k}\right]\;{\left(z_{k}+x\tan\theta \right)}^{3}-{\left(z_{k-1}+x\tan\theta \right)}^{3}$$

(A4) $$[Q]=[T_{\sigma^{\prime }\theta }][T_{\sigma^{\prime \prime }\varphi }][Q^{\prime \prime }]{\left[T_{\sigma^{\prime \prime }\varphi }\right]}^{T}{\left[T_{\sigma^{\prime }\theta }\right]}^{T}$$

(A5) $$[Q^{\prime \prime }]=\left[\begin{array}{ccc} Q_{11}^{\prime \prime } & Q_{12}^{\prime \prime } & 0\\ Q_{12}^{\prime \prime } & Q_{22}^{\prime \prime } & 0\\ 0 & 0 & Q_{66}^{\prime \prime } \end{array}\right]$$

(A6) $$[T_{\sigma \varphi }]=\left[\begin{array}{ccc} \cos^{2}\varphi & \sin^{2}\varphi & -2\cos\varphi \sin\varphi \\ \sin^{2}\varphi & \cos^{2}\varphi & 2\cos\varphi \sin\varphi \\ \cos\varphi \sin\varphi & -\cos\varphi \sin\varphi & \cos^{2}\varphi -\sin^{2}\varphi \end{array}\right]$$

(A7) $$[T_{\sigma \theta }]=\left[\begin{array}{ccc} \cos^{2}\theta & 0 & 0\\ 0 & 1 & 0\\ 0 & 0 & \cos\theta \end{array}\right]$$

where ‘e’ denotes the total number of plies in the laminate. The parameters $z_{k}$ and $z_{k-1}$ represent the coordinates of the top and bottom surfaces of the *k*th lamina layer measured from the center line of the composite plate. The parameters +θ and −θ correspond to the taper angles of the bottom and top surfaces of the composite plate, respectively.

The matrix [Q] represents the reduced stiffness matrix of the ply in the material coordinate system aligned with the fiber and transverse directions. The matrix $\left[Q^{\prime \prime }\right]$ is the reduced stiffness matrix of an individual ply defined along the fiber direction and the two transverse directions. The components of the reduced stiffness matrix $\left[Q^{\prime \prime }\right]$ are given as $Q_{11}^{\prime \prime }=E_{1}/(1-\nu_{12}\nu_{21})$, $Q_{12}^{\prime \prime }=\nu_{12}E_{2}/(1-\nu_{12}\nu_{21})$, $Q_{22}^{\prime \prime }=E_{2}/(1-\nu_{12}\nu_{21})$, $Q_{66}^{\prime \prime }=G_{12}$. ν_12_ and ν_21_ are the corresponding Poisson’s ratios, and G_12_ is the in-plane shear modulus in the material coordinate system. The stress transformation matrix $\left[T_{\sigma \;\varphi }\right]$ transforms stresses from the material coordinate system to the oblique coordinate system by rotating the ply through the fiber orientation angle φ about the *z*-axis. Similarly, the stress transformation matrix $\left[T_{\sigma \theta }\right]$ transforms stresses from the oblique coordinate system to the global x–y coordinate system by rotating the structure through the taper angle θ about the *y*-axis.

In the finite element formulation [42,66], a four-noded rectangular element is employed, with five degrees of freedom assigned to each node. These degrees of freedom consist of $u^{0}$ and $v^{0}$, representing the in-plane displacements of the composite plate along the *x-* and *y*-directions, respectively; $w^{0}$, denoting the transverse deflection at any section of the plate; and $\theta_{x}=\partial w^{0}/\partial y$ and $\theta_{y}=\partial w^{0}/\partial x$, which correspond to the rotational displacements about the *x-* and *y*-axes, respectively. Accordingly, the displacement field at any point within the finite element can be expressed in matrix form as follows.

(A8) $$\left\{\begin{array}{c} u^{0}\\ v^{0}\\ w^{0}\\ \frac{\partial w^{0}}{\partial x}\\ \frac{\partial w^{0}}{\partial y} \end{array}\right\}=\sum_{j=1}^{4}\left[\begin{array}{ccccc} N_{j} & 0 & 0 & 0 & 0\\ 0 & N_{j} & 0 & 0 & 0\\ 0 & 0 & N_{w_{j}^{0}} & N_{\theta yj} & N_{\theta xj}\\ 0 & 0 & \frac{\partial N_{w_{j}^{0}}}{\partial x} & \frac{\partial N_{\theta yj}}{\partial x} & \frac{\partial N_{\theta xj}}{\partial x}\\ 0 & 0 & \frac{\partial N_{w_{j}^{0}}}{\partial y} & \frac{\partial N_{\theta yj}}{\partial y} & \frac{\partial N_{xj}}{\partial y} \end{array}\right]\left\{\begin{array}{c} \;u_{j}^{0}\\ \;v_{j}^{0}\\ \;w_{j}^{0}\\ -\theta_{yj}\\ \;\theta_{xj} \end{array}\right\}$$

$\left\{u\}\;=[N_{j}(x,y)]\{q\right\}$, where j=1,2,3,4.

The relation between strain and displacement field of the element is presented as:

(A9) $$\left\{\chi \}\;=[\bar{B}]\{q\right\}$$

where {χ}, $\left[\bar{B}\right]$, and {q} denote strain vector, strain–displacement matrix, and displacement vector, respectively.

In this formulation, $\{\chi \}={\left\{\frac{\partial u^{0}}{\partial x}\frac{\partial v^{0}}{\partial y}\frac{\partial u^{0}}{\partial y}+\frac{\partial v^{0}}{\partial x}-\frac{\partial^{2}w^{0}}{\partial x^{2}}-\frac{\partial^{2}w^{0}}{\partial y^{2}}-2\frac{\partial^{2}w^{0}}{\partial x\partial y}\right\}}^{T}$ denotes the strain vector of the element, while $\{q\}={\left\{u_{1}^{0},v_{1}^{0},w_{1}^{0},-\theta_{y1},\theta_{x1}\ldots \;u_{4}^{0},v_{4}^{0},w_{4}^{0},-\theta_{y4},\theta_{x4}\right\}}^{T}\;$ represents the corresponding displacement vector. $\left[\bar{B}\right]$ is the strain–displacement matrix, as detailed in Appendix A. The matrices $N_{w_{j}^{0}}$, $N_{\theta y\;j}$, and $N_{\theta x\;j}$ represent the shape functions of the four-noded rectangular element and are provided in Appendix A. The parameters ‘a’ and ‘b’ correspond to the half-length and half-width of the rectangular element, respectively, and $\left(x_{j},y_{j}\right)$ denote the nodal coordinates of the element for *j* = 1, 2, 3 and 4.

The element stiffness, Coriolis, and mass matrices are obtained by substituting Equations (7) and (8) into the strain energy and kinetic energy expressions, as presented below.

(A10) $$[k_{m,b}^{e}]=\int_{-b}^{b}\int_{-a\;}^{a}{\left[{\bar{B}}_{j}(x,y)\right]}^{T}\;\left[\begin{array}{cc} A(x) & B(x)\\ B(x) & D(x) \end{array}\right]\;[{\bar{B}}_{j}(x,y)]\;dxdy$$

(A11) $$[k_{r}^{e}]\;=\int_{-b}^{b}\int_{-a}^{a}{\left[F_{j}(x,y)\right]}^{T}\;\left[\begin{array}{cc} N_{x}\left(x\right) & 0\\ 0 & N_{y}(x) \end{array}\right][F_{j}(x,y)]\;dxdy\;$$

(A12) $$[m^{e}]=\int_{-b}^{b}\int_{-a}^{a}{\left[N_{j}(x,y)\right]}^{T}[I][N_{j}(x,y)]\;dxdy$$

(A13) $$[c^{e}]=\int_{-b}^{b}\int_{-a}^{a}{\left[N_{j}(x,y)\right]}^{T}[I_{1}][N_{j}(x,y)]\;dxdy$$

(A14) $$[k_{a}^{e}]=\int_{-b}^{b}\int_{-a}^{a}{\left[N_{j}(x,y)\right]}^{T}[I_{2}][N_{j}(x,y)]\;dxdy$$

In this expression, matrices $\left[k_{m,b}^{e}],[k_{r}^{e}\right]$ and $\left[k_{a}^{e}\right]$ denote the element linear stiffness matrix associated with membrane and bending deformations, the element stiffness matrix arising from initial stress resultants due to steady-state rotation, and the element stiffness matrix corresponding to the vibratory displacements of the four-noded rectangular plate element, respectively. Matrices $\left[{\bar{B}}_{j}(x,y)\right]$ and $\left[F_{j}(x,y)\right]$ represent the strain–displacement matrices, while [I], $\left[I_{1}\right]$, and $\left[I_{2}\right]$ correspond to the inertia coefficient matrices. The parameters ‘a’ and ‘b’ are the half-length and half-width of the rectangular element, respectively. The governing equations of motion for the tapered composite plate can be formulated in finite element form as:

(A15) $$\left[m^{e}]\{\ddot{q}\;\}+[c^{e}]\{\dot{q}\;\}+[k^{e}]\{q\}=\{f^{e}\right\}$$

Here, $\left[k^{e}]=[k_{m,b}^{e}]+[k_{r}^{e}]+[k_{a}^{e}\right]$, {q} denotes the displacement vector of the rectangular element, and $\left\{f^{e}\right\}$ represents the corresponding element force vector. By assembling the element mass and stiffness matrices together with the force vectors of all elements using MATLAB^®^ software, the global governing equations of motion of the rotating tapered composite plate are obtained in finite element form, which can be written as:

(A16) $$\left[M]\{\ddot{d}\}+[C]\{\dot{d}\}+[K]\{d\}=\{F\right\}$$

In this formulation, [M], [C], and [K] denote the global mass, Coriolis, and stiffness matrices, respectively, while {F} represents the corresponding force vector. In the above equation, the Coriolis matrix [C] is skew-symmetric, whereas the mass and stiffness matrices [M] and [K] are symmetric.

**Table A1:** Effects of variation in volume fraction of graphene on natural frequencies for FO-1 and 750 rpm.

Mode (m,n)	Natural Frequency (Hz)				
	0%	2.5%	5%	7.5%	10%
(1,1)	62.46	65.58	68.23	70.99	72.59
(1,2)	138.55	120.53	125.42	123.97	128.73
(2,1)	312.53	356.00	343.80	357.79	345.42
(2,2)	331.31	389.78	392.95	393.86	397.21

**Table A2:** Effects of variation in volume fraction of graphene on natural frequencies for FO-1 and 1500 rpm.

Mode (m,n)	Natural Frequency (Hz)				
	0%	2.5%	5%	7.5%	10%
(1,1)	61.45	69.40	74.39	79.20	83.92
(1,2)	102.51	113.58	131.53	146.88	160.52
(2,1)	264.52	277.28	320.80	351.36	374.90
(2,2)	359.81	315.56	347.41	380.70	413.37

**Table A3:** Effects of variation in volume fraction of graphene on damping ratio for FO-1 and 750 rpm.

Mode (m,n)	Damping Ratio				
	0%	2.5%	5%	7.5%	10%
(1,1)	0.00298	0.00284	0.00300	0.00250	0.00264
(1,2)	0.00746	0.00984	0.01049	0.00939	0.01003
(2,1)	0.00706	0.01098	0.01126	0.01067	0.01107
(2,2)	0.01815	0.01693	0.01790	0.01680	0.01762

**Table A4:** Effects of variation in volume fraction of graphene on damping ratio for FO-1 and 1500 rpm.

Mode (m,n)	Damping Ratio				
	0%	2.5%	5%	7.5%	10%
(1,1)	0.00308	0.00230	0.00260	0.00294	0.00330
(1,2)	0.00841	0.00560	0.00698	0.00840	0.00990
(2,1)	0.00844	0.00614	0.00866	0.01090	0.01278
(2,2)	0.01858	0.00978	0.01000	0.01031	0.01116

**Table A5:** Effects of variation in volume fraction of MWCNT on natural frequency for FO-1 and 750 rpm.

Mode (m,n)	Natural Frequency (Hz)				
	0%	2.5%	5%	7.5%	10%
(1,1)	62.46	69.877	71.59	73.03	75.05
(1,2)	138.55	181.00	182.64	184.29	185.99
(2,1)	312.53	424.79	438.53	447.67	458.84
(2,2)	331.31	447.24	468.28	487.80	512.66

**Table A6:** Effects of variation in volume fraction of MWCNT on natural frequency for FO-1 and 1500 rpm.

Mode (m,n)	Natural Frequency (Hz)				
	0%	2.5%	5%	7.5%	10%
(1,1)	61.45	74.95	76.54	77.90	79.80
(1,2)	102.51	182.93	184.55	186.19	187.87
(2,1)	264.52	426.72	441.30	450.76	462.02
(2,2)	359.81	450.22	470.27	489.37	513.99

**Table A7:** Effects of variation in volume fraction of MWCNT on damping ratio for FO-1 and 750 rpm.

Mode (m,n)	Damping Ratio				
	0%	2.5%	5%	7.5%	10%
(1,1)	0.00298	0.00242	0.00261	0.00278	0.00302
(1,2)	0.00746	0.01332	0.01365	0.01394	0.01427
(2,1)	0.00706	0.01754	0.01782	0.01839	0.01920
(2,2)	0.01815	0.01775	0.01808	0.01827	0.01865

**Table A8:** Effects of variation in volume fraction of MWCNT on damping ratio for FO-1 and 1500 rpm.

Mode (m,n)	Damping Ratio				
	0%	2.5%	5%	7.5%	10%
(1,1)	0.00308	0.00223	0.00241	0.00257	0.00280
(1,2)	0.00841	0.01309	0.01343	0.01372	0.01404
(2,1)	0.00844	0.01743	0.01759	0.01812	0.01892
(2,2)	0.01858	0.01755	0.01801	0.01824	0.01864

**Table A9:** Effects of variation in volume fraction of fullerene on natural frequency for FO-1 and 750 rpm.

Mode (m,n)	Natural Frequency (Hz)				
	0%	2.5%	5%	7.5%	10%
(1,1)	62.46	63.97	64.93	65.59	67.82
(1,2)	138.55	124.29	120.18	125.09	120.25
(2,1)	312.53	343.24	355.88	343.68	332.74
(2,2)	331.31	391.53	389.67	392.84	425.86

**Table A10:** Effects of variation in volume fraction of fullerene on natural frequency for PLO-1 and 1500 rpm.

Mode (m,n)	Natural Frequency (Hz)				
	0%	2.5%	5%	7.5%	10%
(1,1)	61.45	65.58	68.23	70.99	72.59
(1,2)	102.51	120.53	125.42	123.97	128.73
(2,1)	264.52	356.00	343.80	357.79	345.42
(2,2)	359.81	389.78	392.95	393.86	397.21

**Table A11:** Effects of variation in volume fraction of fullerene on damping ratio for PLO-1 and 750 rpm.

Mode (m,n)	Damping Ratio				
	0%	2.5%	5%	7.5%	10%
(1,1)	0.00298	0.00164	0.00290	0.00306	0.00316
(1,2)	0.00746	0.00837	0.00990	0.01054	0.01065
(2,1)	0.00706	0.01168	0.01099	0.01126	0.01133
(2,2)	0.01815	0.01066	0.01694	0.01791	0.01799

**Table A12:** Effects of variation in volume fraction of fullerene on damping ratio for PLO-1 and 1500 rpm.

Mode (m,n)	Damping Ratio				
	0%	2.5%	5%	7.5%	10%
(1,1)	0.00308	0.00250	0.00264	0.00284	0.00300
(1,2)	0.00841	0.00939	0.01003	0.00984	0.01049
(2,1)	0.00844	0.01067	0.01107	0.01098	0.01126
(2,2)	0.01858	0.01680	0.01763	0.01693	0.01790
