# Supplementary material for: Dynamics of Drone Blades Based on Polymer Nanocomposites Incorporating Graphene, Carbon Nanotube, and Fullerene
Source: Polymers (Basel). 2026 Mar 23;18(6):778. doi: 10.3390/polym18060778 (PMC13030742; doi:10.3390/polym18060778)
Supplement: Supplementary file 1 [file polymers-18-00778-s001.zip › polymers-4168613-supplementary.pdf]

Supplementary Materials

Dynamics of Drone Blades Based on Polymer Nanocomposites Incorporating Graphene, Carbon Nanotube, and Fullerene

To ensure full reproducibility of the numerical simulation described in this study—which investigates the dynamic properties (natural frequencies, damping ratios, and mode shapes) of tapered aramid (Kevlar)/epoxy nanocomposite drone blades reinforced with nanocarbons (graphene, MWCNT, and fullerene)—comprehensive modeling information is provided below. This includes details on geometry, material properties, modeling approaches, boundary and loading conditions, parametric ranges, and software specifications.

1. Geometry and Structural Configuration

Blade model: Tapered laminated composite plate with linear taper along the longitudinal direction x.

Dimensions:

Length (L, span): 300 mm; Width (chord): 200 mm; Root thickness (at clamped end, left): 13 mm; Tip thickness (free end, right): 8 mm.

Taper implementation: The plate is divided into S domains (number of sections along x) to approximate the linearly varying thickness due to ply drop-offs.

Laminate construction:

Top and bottom face sheets: Each consists of three plies (each ply thickness = 1.25 mm), for a total face sheet thickness of 3.75 mm per side.

Central core: Single honeycomb layer (array of hexagonal pillars), providing the majority of the thickness variation.

Overall sandwich-like structure with ply drop-offs to achieve taper.

2. Material Properties

Kevlar 49 aramid fiber (unidirectional):

Density (ρ): 1.44 g/cm³; Longitudinal modulus (E_1): 112–124 GPa (commonly 112–124 GPa); Transverse modulus (E_2): ~2–8 GPa; Shear modulus (G_{12}): ~2–5 GPa; Poisson's ratio (ν_{12}): 0.34–0.36; Filament diameter: 12 μm.

Epoxy matrix (baseline):

Density (ρ): 1.1–1.2 g/cm³; Young's modulus (E): 3.0–4.0 GPa; Poisson's ratio (ν): 0.28–0.35

Kevlar/epoxy unidirectional lamina (typical $V_f \approx$ 50–60%):

E_1: 60–80 GPa; E_2: 4–10 GPa; G_{12}: 2–5 GPa; ν_{12}: 0.3–0.45; ρ: 1.3–1.4 g/cm³

Honeycomb core (aramid/Nomex-like, hexagonal):

Density (ρ): 0.03–0.08 g/cm³; Through-thickness modulus (E_3): 0.1–1 GPa; Shear moduli: 20–100 MPa

Nanofiller-reinforced matrix (0–10 wt.% in epoxy; effective properties via rule-of-mixtures or Halpin-Tsai): Orthotropic reduced stiffness matrix [Q] for each ply is derived from the above using classical laminate theory.

3. Modeling and Numerical Method

Theoretical basis: *Classical Laminate Plate Theory* (CLPT) extended for tapered plates, incorporating geometric nonlinearity, rotational effects (centrifugal stiffening, Coriolis), and shear deformation where applicable.

Numerical approach: *Finite Element Method* (FEM).

Discretization: Domain divided into finite elements to capture variable thickness, ply drop-offs, anisotropic properties, and discontinuities.

Matrices assembled: Stiffness matrix, mass matrix, Coriolis matrix (for rotation), geometric stiffness (if nonlinear).

Eigenvalue problem solved for natural frequencies and mode shapes.

Damping ratio: Incorporated (likely modal or Rayleigh damping; trends reported per mode and nanofiller).

Boundary conditions: *Clamped-Free-Free-Free* (CFFF)

4. Parametric Ranges Studied

Nanofiller weight fraction: 0–10 wt.% (key results at 5 wt.%).

Angular (rotational) speed: 750–2250 rpm (key at 1500 rpm medium speed).

Setting angle (twist/pitch angle, φ): 30°–60° (key at 45°).

Fiber orientation in plies: Varied (specific orientations labeled 1, 2, etc.; mode-dependent trends).

Nanofillers compared: Graphene (2D), MWCNT (1D), fullerene (0D).

5. Software and Implementation

Software: MATLAB (2016 version, specifically R2016a release).

Implementation: Custom scripts (no specialized toolboxes required for core FEM; possibly basic functions for matrix operations and eigensolvers).

Assembly of stiffness, mass, and Coriolis matrices (see A8–A14 in Appendix).

Parametric loops for variations in nanofiller wt.%, rpm, setting angle, fiber orientation.

Eigenvalue solution for natural frequencies: post-processing for damping ratios (likely from complex eigenvalues or assumed models) and mode shapes.

Validation note: Analytical solutions infeasible due to taper, rotation, and nanofiller effects; numerical convergence assumed via element refinement.

6. The Properties and Dimension of Fibers

These properties are cited from the official DuPont Kevlar® Aramid Fiber Technical Guide (revised versions, e.g., 2019 and earlier equivalents),[S1] which is the primary manufacturer reference and most commonly used source in composite modeling and research:

Density (ϱ): 1.44 g/cm^3

(DuPont Kevlar® Technical Guide, Table II-1 and comparative properties table)

Filament diameter: 12 μm.

DuPont Kevlar® Technical Guide, footnote to Table II-1: "Filament diameter is 12 μm."

Tensile strength: 3,000–3,600 MPa (yarn: ~3,000 MPa; resin-impregnated strands: up to 3,600 MPa)

DuPont Kevlar® Technical Guide, Table II-1: 435,000 psi (3,000 MPa) for yarn; 525,000 psi (3,600 MPa) for impregnated strands; also consistent in Jones, 1983 DTIC report ADA136614.

Young's modulus (tensile modulus): 112–124 GPa (yarn: ~112 GPa or 16.3 × 10^6 psi; impregnated strands: up to 124 GPa or 18.0 × 10^6 psi)

Note that this study used unidirectional (UD) plies. The research describes the parametric variation of "fiber orientation in laminate layers" (e.g., "fiber orientation 1" vs. "fiber orientation 2"), noting mode-dependent trends in natural frequency and damping. The top and bottom layers are composed of discrete plies—three plies each, at 1.25 mm per ply—with explicit orientation effects. This is characteristic of UD laminates, where the stacking sequence and fiber angle control anisotropy ($E_1 >> E_2$). The focus on directional sensitivity matches the behavior of UD laminates commonly used in aerospace and drone blades for spanwise and chordwise optimization, particularly in tapered structures with ply drop-offs.

References

S1. DuPont de Nemours, Inc. Kevlar® Aramid Fiber Technical Guide. DuPont: Wilmington, DE, USA, 2019; Table II-1 and Comparative Properties Table. Available online: https://www.dupont.com/content/dam/dupont/amer/us/en/safety/public/documents/en/Kevlar_Technical_Guide_0319.pdf
